# Cholesterol Changes Interfacial Water Alignment in Model Cell Membranes

**DOI:** 10.1021/jacs.4c00474

**Published:** 2024-04-30

**Authors:** Hanna Orlikowska-Rzeznik, Jan Versluis, Huib J. Bakker, Lukasz Piatkowski

**Affiliations:** † Faculty of Materials Engineering and Technical Physics, Poznan University of Technology, 60-965 Poznan, Poland; ‡ AMOLF, Ultrafast Spectroscopy, 1098 XG Amsterdam, The Netherlands

## Abstract

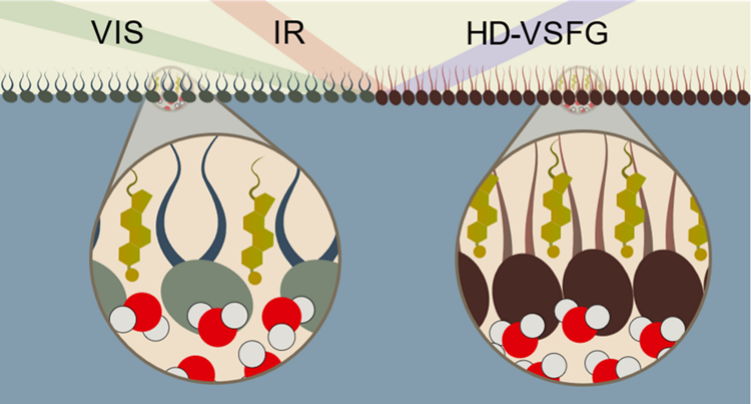

The nanoscopic layer of water that directly hydrates biological membranes plays a critical role in maintaining the cell structure, regulating biochemical processes, and managing intermolecular interactions at the membrane interface. Therefore, comprehending the membrane structure, including its hydration, is essential for understanding the chemistry of life. While cholesterol is a fundamental lipid molecule in mammalian cells, influencing both the structure and dynamics of cell membranes, its impact on the structure of interfacial water has remained unknown. We used surface-specific vibrational sum-frequency generation spectroscopy to study the effect of cholesterol on the structure and hydration of monolayers of the lipids 1,2-dipalmitoyl-*sn*-glycero-3-phosphocholine (DPPC), 1,2-dioleoyl-*sn*-glycero-3-phosphocholine (DOPC), and egg sphingomyelin (SM). We found that for the unsaturated lipid DOPC, cholesterol intercalates in the membrane without significantly changing the orientation of the lipid tails and the orientation of the water molecules hydrating the headgroups of DOPC. In contrast, for the saturated lipids DPPC and SM, the addition of cholesterol leads to clearly enhanced packing and ordering of the hydrophobic tails. It is also observed that the orientation of the water hydrating the lipid headgroups is enhanced upon the addition of cholesterol. These results are important because the orientation of interfacial water molecules influences the cell membranes’ dipole potential and the strength and specificity of interactions between cell membranes and peripheral proteins and other biomolecules. The lipid nature-dependent role of cholesterol in altering the arrangement of interfacial water molecules offers a fresh perspective on domain-selective cellular processes, such as protein binding.

## Introduction

The life of eukaryotic cells relies critically on the presence and properties of lipid membranes. First, they maintain the cellular integrity by separating the cell interior from the extracellular milieu and by defining the subcellular organelles.^1^ Second, they create a dynamic molecular matrix that supports the vital functions of integral membrane proteins,^2^ thereby facilitating a wide range of biochemical processes, including neurotransmission,^3^ energy production,^4^ and immune response.^5^ Cell membranes exhibit a huge molecular heterogeneity of lipid compounds, as evident in the ever-evolving field of lipidomics, which aims to identify and quantify the molecular species of cellular lipids and their biological functions.^6^ Yet, it has been long recognized that the most abundant lipids found in mammalian cell membranes can be generally divided into three categories: glycerophospholipids (phosphatidylcholines in particular), sphingolipids, and cholesterol, which along with the nanoscopic layer of water directly hydrating the lipids, commonly referred to as *biological water*, define the structural scaffold of cellular membranes.^7^

The properties of biological water differ markedly from water in the bulk due to confinement effects and a perturbed interfacial H-bond donor/acceptor balance.^8−10^ Interfacial water is characterized by greatly slowed down rotational^11^ and translational^12^ dynamics as a result of the formation of strong hydrogen bonds with the polar moieties of lipids (e.g., phosphate and carbonyl). Lipid−water interactions also affect the structure of interfacial water. Strong evidence has been found that biological water is highly polarized such that the hydrogen atoms point toward the membrane interior in the case of zwitterionic phospholipids.^13−18^ The preferential arrangement of the lipid moieties and the nonrandom orientation of the associated water molecules determine the sign and magnitude of the membrane dipole potential,^19^ which was shown to strongly influence a variety of membrane-centered processes, such as drug binding,^20^ translocation of ions and macromolecules,^21,22^ clustering and binding affinity of proteins^23^ as well as the function of membrane-incorporated proteins,^24^ and many more.^25−27^ Furthermore, the molecular structure of biological water has been postulated to dictate the fusogenic properties of lipid membranes.^28^ In a molecular dynamics simulation study by Kasson et al., the ordering of water bound to the surface of biomembranes was found to control membrane fusion dynamics.^29^ Consequently, any alteration in the arrangement of interfacial water has potential impact on all processes in which membrane fusion plays a fundamental role, such as neurotransmission, fertilization, viral entry, exocytosis, and intracellular transport.^30^

The existing literature provides valuable insights into the structure and dynamics of biological water, yet it is important to note that these findings are primarily derived from studies involving pure phospholipid model systems. Given that cholesterol is a fundamental component of mammalian cell membranes and that it plays a crucial role in regulating both the structure and dynamics of lipid membranes^31^ as well as functioning of membrane proteins,^32^ its influence on both the dynamics and structural aspects of biological water should be explicitly acknowledged. This gains further importance considering that cholesterol, along with sphingomyelin, is associated with the formation of so-called lipid rafts,^33^ i.e., transient functional domains involved in various cellular processes such as membrane trafficking, signal transduction, or host−pathogen interactions.^34,35^

It is well documented that cholesterol’s interactions with adjacent phospholipids induce conformational ordering of lipid alkyl chains, reduce the area per lipid, and increase the thickness of the lipid bilayer,^36−38^ thereby changing the permeability^39^ and mechanical properties of the membrane.^40^ In contrast, the effect of cholesterol on interfacial water properties remains largely unexplored experimentally. Cheng et al. using ^1^H Overhauser dynamic nuclear polarization relaxometry found that cholesterol enhances the translational diffusivity of water at the surface of the phosphatidylcholine bilayer and attributed this finding to potential weakening or breaking of the strong hydrogen-bond network of the surface hydration layers.^41^ Recently, Pyne et al., by employing attenuated total reflection–Fourier transform infrared spectroscopy in the terahertz frequency domain, found evidence that cholesterol weakens the interfacial intermolecular hydrogen bonds at a lipid bilayer composed of negatively charged lipids, which eventually leads to accelerated global dynamics of water at the membrane.^42^ However, they found a negligible effect in the case of zwitterionic lipids.

These important consequences of the intimate interactions of cholesterol with the membrane prompt the question whether changes in the lipid structure and dynamics, along with affected interfacial water dynamics, are accompanied by alterations in the arrangement of biological water molecules. In a quest to gain a more detailed picture of the potential impact of cholesterol on the orientation of water molecules, which plays a pivotal role in determining the membrane dipole potential, we pose several questions: Does cholesterol influence membrane-bound water ordering? If so, to what extent, and how does this effect depend on the nature of the lipid matrix? Finally, what mechanisms are at play—does cholesterol in the membrane interact with water directly, or it exerts its effects indirectly by altering the arrangement of adjacent phospholipids?

To study how cholesterol affects the hydrogen-bonding network of biological water, we employed heterodyne-detected vibrational sum-frequency generation (HD-VSFG) spectroscopy, which is inherently an interface-specific technique, using zwitterionic lipid monolayers as a model for cell membranes. By altering the cholesterol content of the membrane, we explored its impact on the hydration characteristics of three types of phospholipids: two phosphatidylcholines (unsaturated and saturated) and sphingomyelin. The interface was probed in two vibrational regions: The first region covers the bending vibrations of lipid methyl and methylene groups and the stretch vibration of lipid carbonyls, providing information on the lipid structure; the second region covers the stretching of lipid methyl and methylene groups, along with the stretch vibration of the water hydroxyl groups, the latter providing direct insight into the structure of the interfacial water. In the context of membrane hydration structure, phospholipid carbonyls hold particular significance as they terminate the hydrogen-bond network of biological water hydrating the membrane.^43^ This is due to their spatial positioning within the boundary region, which separates the well-solvated polar lipid heads from the hydrophobic lipid fatty acid chains. By integrating water-centric and lipid-centric viewpoints, we were able to correlate the changes induced by cholesterol on the membrane structure with alterations in the structural arrangement of water molecules within the phospholipid headgroups.

## Materials and Methods

### Materials

**Figure 1:**
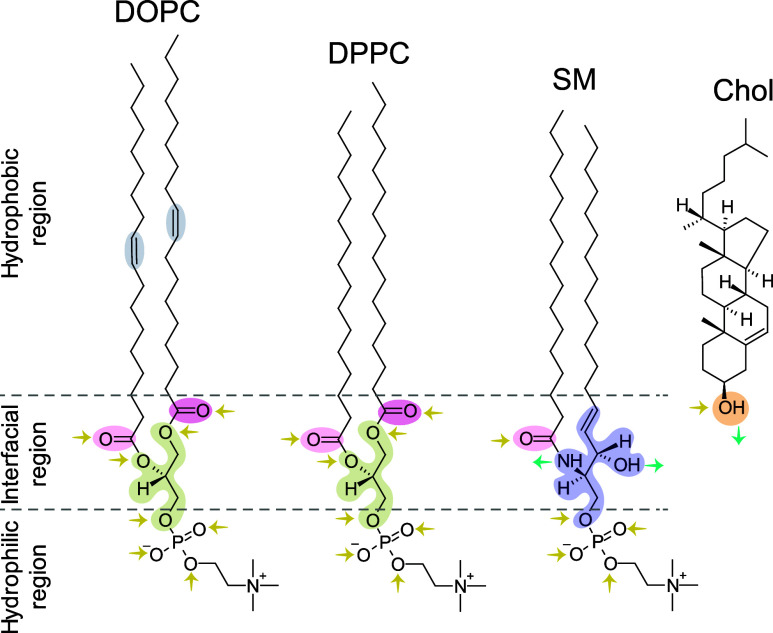
Molecular structures of the studied lipids: DOPC, DPPC, SM, and Chol. The glycerol linkage of phosphatidylcholines (DOPC, DPPC) is indicated in green. The sphingosine linkage of SM is indicated in blue. Carbonyl groups are indicated in pink shades. The hydroxyl group of cholesterol is indicated in orange. Double bonds in the hydrocarbon tails of DOPC are indicated in gray. Hydrogen-bond accepting and donating atoms are indicated by yellow and turquoise arrows, respectively.

Lipids 1,2-dipalmitoyl-*sn*-glycero-3-phosphocholine (DPPC), 1,2-dioleoyl-*sn*-glycero-3-phosphocholine (DOPC), egg sphingomyelin (SM), and cholesterol (Chol) were supplied by Avanti Polar Lipids. D_2_O (99.9 atom % D) and spectrophotometric grade chloroform were purchased from Sigma-Aldrich. All compounds were used as received without further purification. The concentration of phospholipid stock solutions in chloroform was 0.1 mM. Deionized water was acquired using the Millipore Nanopure system (18.2 MΩ cm, pH 5.5). The molecular structures of the studied lipids, Chol and the three zwitterionic phospholipids DOPC, DPPC, and SM, are depicted in Figure 1. The structural differences between the lipids are highlighted.

### Sample Preparation

Following a thorough cleaning procedure with ethanol and ultrapure water, a home-built Teflon trough (35 mm diameter) was filled with either H_2_O or D_2_O as a subphase. H_2_O was used for measurements in the CH and OH stretching vibration regions. D_2_O was used for measurements in the CH bending and CO stretching vibration regions to avoid the measurements being affected by the response of the water bending mode. Self-assembled lipid monolayers were formed by dropwise spreading a lipid stock solution onto the surface of the neat subphase using a microliter Hamilton syringe. After spreading, at least 1 min was allowed for solvent evaporation and monolayer equilibration before sum-frequency generation spectroscopy measurements were taken. Lipid surface coverage (surface pressure) was controlled by the amount of lipid solution applied onto the subphase and was kept constant throughout the experiment. The HD-VSFG data presented herein correspond to the case where the surface is fully covered with lipids, indicated by the complete vanishing of the response of unbound water OH groups (see Figure S1). The amount of lipid solution applied onto the subphase to fulfill this condition corresponds to a surface pressure of around 40 mN/m for each sample (pure Chol, pure phospholipids, and phospholipid mixtures with Chol). The surface pressure was measured independently, outside the HD-VSFG experimental setup, using a Langmuir−Blodgett (KSV Nima) balance equipped with a platinum Wilhelmy plate. The measurements were conducted at a temperature of 21 °C. Under these conditions, all of the lipid monolayers were in a condensed-phase state. The studied phospholipids mix effectively with cholesterol in the entire range of the investigated molar fractions.^44−46^

### Heterodyne-Detected Vibrational Sum-Frequency Generation Spectroscopy (HD-VSFG)

HD-VSFG experiments were carried out using a home-built optical setup based on an amplified Ti:sapphire laser system (oscillator: Coherent Mantis, amplifier: Coherent Legend Elite Duo). The laser system produced 35 fs pulses centered at 800 nm, with an energy of approximately 6.5 mJ and a repetition rate of 1 kHz. The laser output was divided into two parts, one of which was used to pump a commercial optical parametric amplifier (Light Conversion HE-TOPAS) to generate tunable infrared (IR) pulses with a spectral bandwidth of ∼400 cm^−1^. The IR pulses were centered at approximately 3200 and 1600 cm^−1^ (with bandwidths at half-maximum of about 550 and 350 cm^−1^) to probe the hydroxyl stretching and phospholipid carbonyl stretching regions, respectively. The other part of the laser output (visible, VIS) underwent spectral narrowing (using diffraction grating and spatial filtering) to a bandwidth of approximately 20 cm^−1^, which largely determines the spectral resolution of the detected SFG signal. The IR and VIS beams were spatially and temporally overlapped at the surface of a gold mirror, leading to the generation of light at their sum frequency. This sum-frequency generation (SFG) signal, originating from the strong nonresonant second-order nonlinear susceptibility (χ^2^) of gold, served as a local oscillator (LO-SFG). The IR and VIS beams had incidence angles of ∼55 and ∼50°, respectively, relative to the surface normal. Before the sample, in the optical path of the reflected LO-SFG signal, a 1 mm thick silica plate was inserted to introduce a time delay of approximately 1.6 ps of the SFG light with respect to the IR and VIS beams. Subsequently, all three beams were focused by a spherical mirror onto the sample surface, where the IR and VIS beams overlapped spatially and temporally and generated a sample vibrational sum-frequency generation (VSFG) signal. The delayed LO-SFG and sample VSFG were subsequently coupled into a spectrograph (Princeton Instruments Acton SpectraPro SP-2300), and the spectral interference pattern of the two light signals was detected using a charge-coupled device (CCD) camera (Princeton Instruments Pixis 100). To extract the phase of the sample VSFG light, we repeated the HD-VSFG experiments using a z-cut quartz crystal instead of the sample. To obtain the extracted phase with sufficient accuracy, the quartz crystal was placed at the same height as the sample, which was controlled by monitoring the position of the signal on the CCD camera. Spectra were collected in ssp polarization combination (s: polarized SFG, s: polarized VIS, p: polarized IR). To minimize the loss of IR intensity caused by water vapor absorption along the optical path and to prevent unsaturated lipid oxidation (DOPC), the optical setup was continuously purged with nitrogen gas.

The measured interference spectra are affected by spectral modulation effects, resulting from an etaloning effect in the CCD camera, which is particularly strong in the 1600 cm^−1^ region (for details see Supporting Information, Note S1). To correct for this effect, we performed two separate measurements on a z-cut quartz crystal, for which crystal orientation differed by 180°. By summing two reference spectra with a 180° phase difference, we effectively eliminated the interference from LO-SFG and the quartz SFG. The remaining modulation primarily represents the structural noise. This remaining signal was then utilized to correct the measured signal from the samples for both the spectral dependence of the input IR beam and the etaloning effect, using the procedure described by Moll et al.^47,48^ The Teflon trough with water and a lipid monolayer was positioned on a rotating holder to facilitate the continuous renewal of the sample-probed area. This approach served two main purposes: (i) to average the HD-VSFG signal across distinct locations on the sample surface and (ii) to mitigate laser-induced thermal effects—specifically, the Marangoni flows resulting from steady-state laser heating, which displace lipids from the laser focal region.^49,50^ In the analysis conducted, the raw interferograms were subjected to Fourier transformation for further processing. The spectra processing was realized using a custom Python script. Each HD-VSFG spectrum presented in the figures is averaged over at least three spectra per membrane composition, unless otherwise indicated.

## Results and Discussion

### Impact of Cholesterol on the Interfacial Monolayer Structure

**Figure 2:**
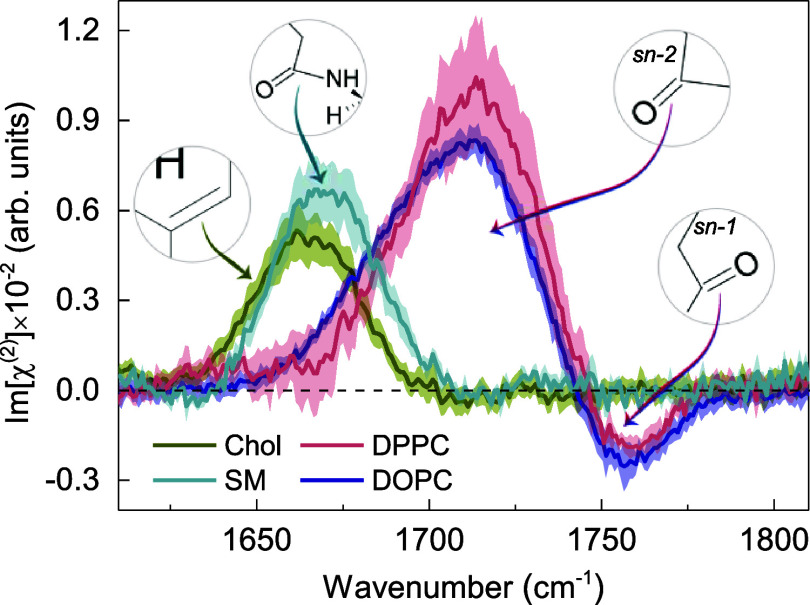
Imχ^(2)^ spectra of Chol, DOPC, DPPC, and SM monolayers at the D_2_O subphase in the phospholipid carbonyl stretching vibration region. Curves represent the mean spectra with shaded areas indicating the standard deviation.

First, we analyze the spectral signatures from the individual membrane constituents in the carbonyl (C=O) stretching region. The direct comparison of the Imχ^(2)^ spectra of pure cholesterol and the cholesterol-free DOPC, DPPC, and SM monolayers at the D_2_O–air interface is depicted in Figure 2.

The Imχ^(2)^ spectrum of the pure cholesterol–D_2_O interface, in the phospholipid carbonyl stretching region, is characterized by a pronounced positive peak at 1665 cm^−1^.

In a recent study of sphingomyelin–cholesterol interactions in lipid monolayers,^51^ the aforementioned band has been overlooked in the intensity VSFG spectrum measured with the same polarization combination as used here (ssp). Following Genova et al.^52^ who reported Raman spectra of the neat cholesterol film, we attribute the band at 1665 cm^−1^ to the carbon-to-carbon double-bond (C=C) stretching vibration from the steroid ring structure.

In the Imχ^(2)^ spectra of both DOPC–D_2_O and DPPC–D_2_O interfaces, two bands of opposite signs and different amplitudes can be distinguished: a prominent positive peak at approximately 1715 cm^−1^ and a weaker negative one at ∼1760 cm^−1^. These two partially overlapping bands are assigned to the phosphatidylcholine ester carbonyl stretching vibrations and have been previously identified by linear infrared spectroscopies.^53,54^ Consistent with previous studies, we assign the lower-frequency band to hydrogen-bonded phospholipid carbonyls and the higher-frequency band to non-hydrogen-bonded carbonyl groups.^53^ The signs of the Imχ^(2)^ responses from the two carbonyl populations suggest that the hydrogen-bonded C=O groups are preferentially oriented with their oxygen atoms pointing toward the bulk water region (O-down), whereas the free C=O groups are preferentially oriented toward the hydrophobic part of the membrane (O-up). This interpretation of the response of the C=O vibrational modes is consistent with the molecular picture presented by Dreier et. al, arising from the analysis of the HD-VSFG data for the monolayers composed of the charged lipids (DPTAP and DPPG).^55^ The positive band likely originates mainly from *sn-2* carbonyls with a net O-down orientation, located closer to the bulk water region and thus hydrated through hydrogen bonding. On the other hand, the negative band most likely arises predominantly from *sn-1* carbonyls, which preferentially orient with their oxygen atoms toward the hydrophobic region of the membrane (O-up), located further away from the bulk water region and, as a result, nonhydrated.

In contrast to phosphatidylcholines (PCs), the Imχ^(2)^ spectrum of the SM–D_2_O interface exhibits only one pronounced band at ∼1670 cm^−1^, which originates from the stretching mode of the carbonyl group in the N-linked acyl chain (amide I).^56^ The positive sign and relatively small width of the band indicates a homogeneous orientational distribution of SM C=O groups with their oxygens atoms pointing toward the bulk water region (O-down). Furthermore, its spectral position (45 cm^−1^ shift to a lower wavenumber with respect to DPPC and DOPC) suggests that these moieties are hydrated. We note here that the amide modes are typically observed at lower frequencies than carbonyl modes from PC lipids.^57^

**Figure 3:**
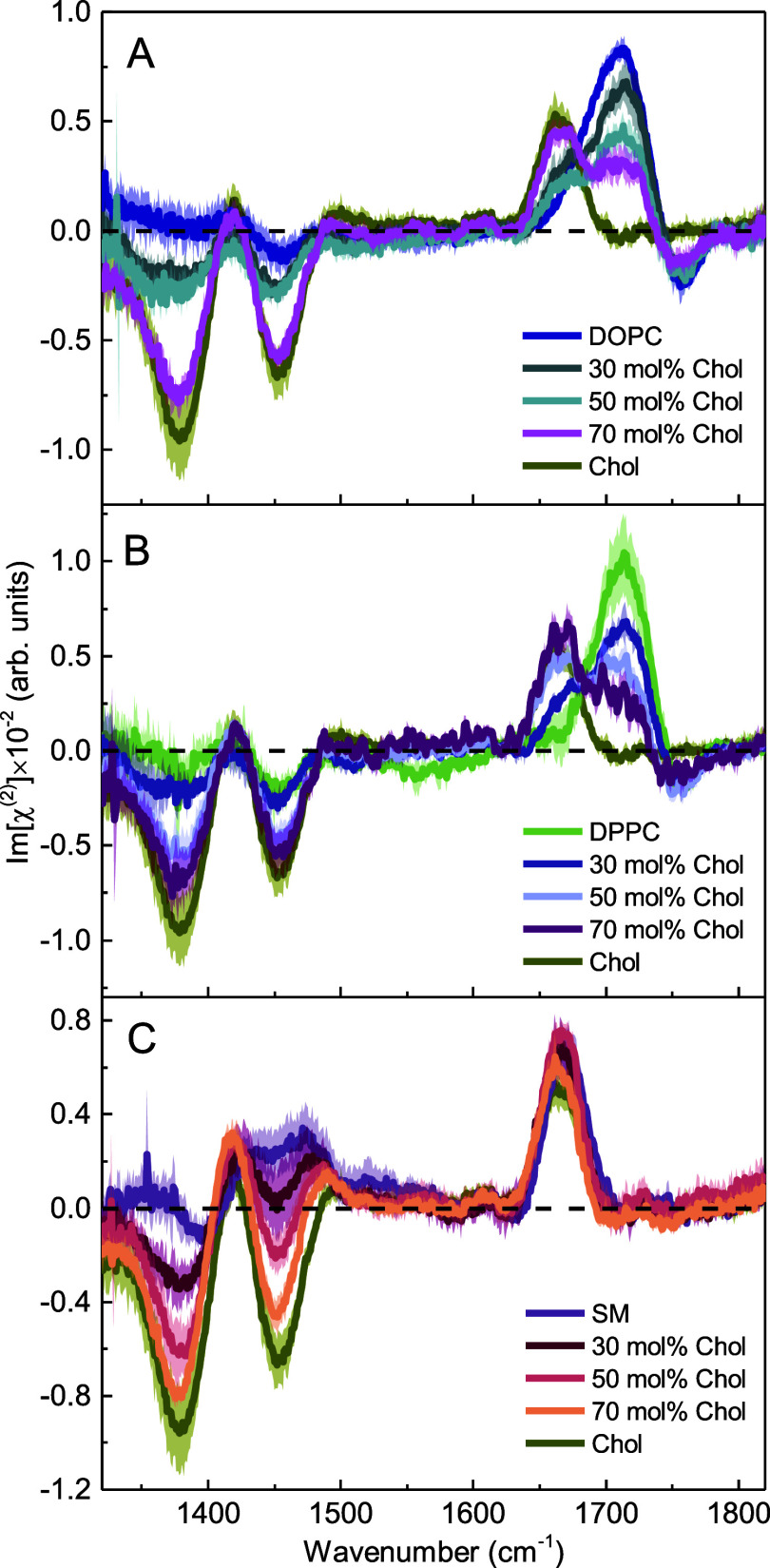
Imχ^(2)^ spectra of (A) DOPC, (B) DPPC, and (C) SM monolayers at the D_2_O subphase in the absence and presence of cholesterol at molar fractions of 0.3, 0.5, and 0.7 in the CH bending and C=O stretching vibration regions. The Imχ^(2)^ spectrum of Chol is shown for reference. Curves represent the mean spectra with shaded areas indicating the standard deviation.

In Figure 3, we present Imχ^(2)^ spectra of the interface of mixed phospholipid/cholesterol monolayers on D_2_O in the CH bending and C=O stretching regions. The Imχ^(2)^ spectra of pure components are shown for reference. The two negative peaks at 1385 and 1460 cm^−1^, found in the Imχ^(2)^ spectra of all mixed monolayers, are ascribed to the methyl and methylene bending modes. In the case of SM, we also observe contributions from the NH bending modes in this spectral region (amide II and/or amide III).^56^

As shown in Figure 3A and B, upon increasing the cholesterol content in the DOPC and DPPC monolayers, the positive and negative bands of the carbonyl stretch vibrations (1715 and 1760 cm^−1^) decrease, while the amplitudes of the bands of the Chol C=C stretching (1665 cm^−1^) and CH bending modes increase.

**Figure 4:**
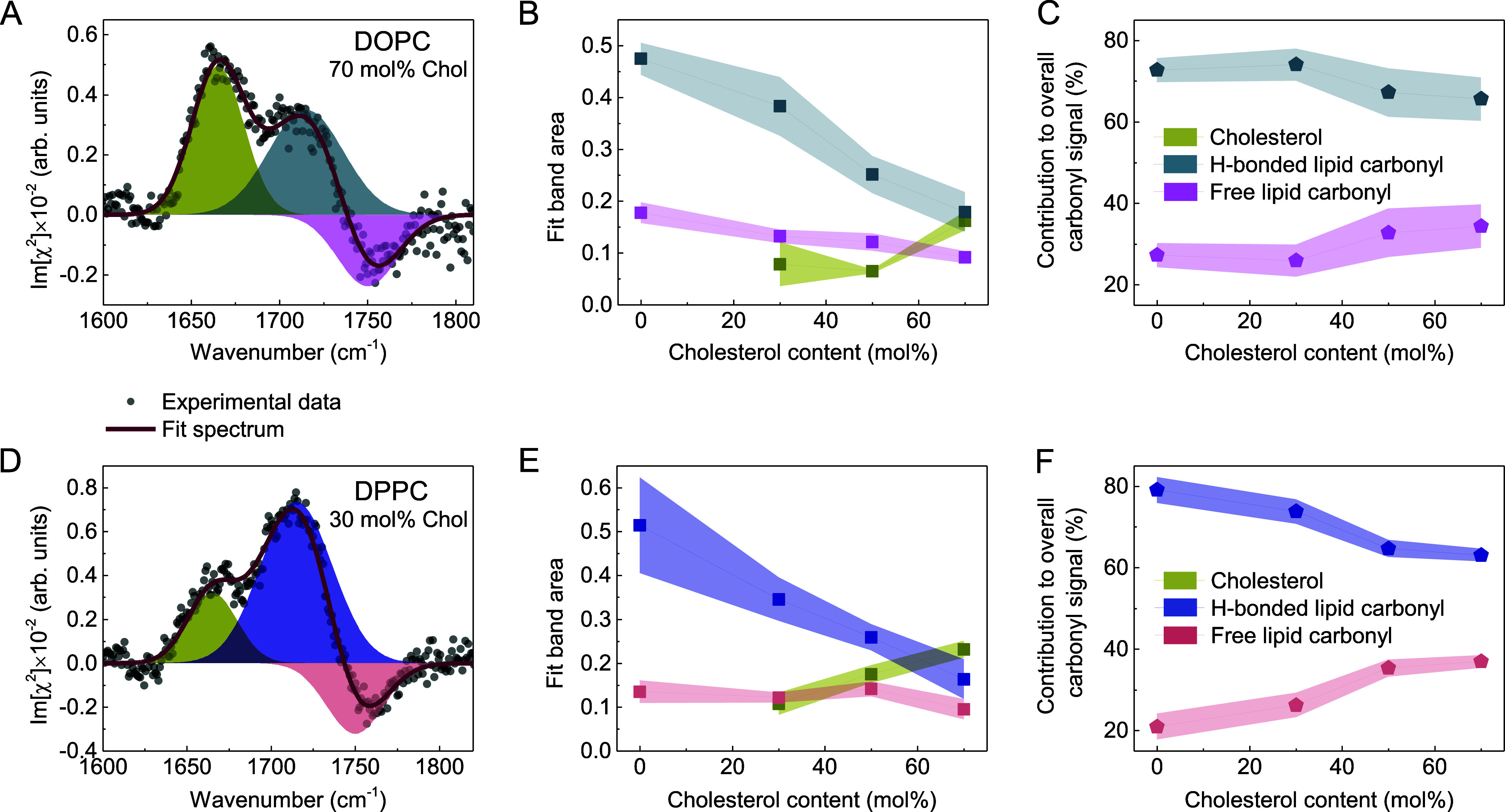
Results of Imχ^(2)^ spectra decomposition using three Gaussian line shapes in the lipid carbonyl stretching region for cholesterol-containing PC lipid monolayers. The upper panels (A–C) correspond to DOPC and the bottom ones (D–F) to DPPC samples. (A, D) Three peak Gaussian decomposition of the exemplary Imχ^(2)^ spectrum of the phospholipid/Chol monolayer. (B, E) The area of the distinct Gaussian components as a function of cholesterol concentration. (C, F) Contribution of the hydrogen-bonded and non-hydrogen-bonded carbonyls to the overall signal from carbonyls, calculated based on the fitted band areas, as a function of cholesterol concentration. Curves on panels B, C, E, and F represent the mean values with shaded areas indicating the standard deviation. The lines connecting the points act as a guide to the eye.

To quantify the effect and to gain a more detailed molecular-level insight into the cholesterol-induced changes at the carbonyl level of the membranes composed of DOPC and DPPC, we performed spectra decomposition using three Gaussian line shapes, to account for the contribution from cholesterol and two oppositely oriented carbonyl populations. The results of this analysis are plotted in Figure 4.

The Imχ^(2)^ spectra of the monolayers composed of DPPC and DOPC enriched with cholesterol can be well expressed as a linear combination of the bands centered at: (i) 1665 cm^−1^ with a width (full width at half-maximum) of 35 cm^−1^, assigned to the C=C stretch vibration from the cholesterol ring structure, (ii) 1715 cm^−1^ with a width of 50 cm^−1^, assigned to the hydrogen-bonded phospholipid carbonyls, and (iii) 1750 cm^−1^ with a width of 40 cm^−1^, attributed to the non-hydrogen-bonded phospholipid carbonyls. The results of the fitting procedure for the Chol-DOPC and Chol-DPPC monolayers at a cholesterol content of 70 and 30 mol % are depicted in Figure 4A and D, respectively.

In Figure 4B and E, it is evident that with an increase in cholesterol content, the areas of both peaks corresponding to distinct carbonyl populations decrease with respect to the pure DOPC and DPPC monolayers. This observation aligns with expectations as the introduction of cholesterol leads to an exchange of a portion of PC lipids, resulting in an increased sterol contribution to the Imχ^(2)^ spectra, accompanied by a simultaneous decrease in the contribution from PC (dilution effect). However, the areas of the two peaks, and therefore the abundance of both detected carbonyl populations, do not diminish at the same rate or to the same extent. Figure 4C and F demonstrates that as the cholesterol content in the membranes increases, the relative contribution of the non-hydrogen-bonded carbonyls to the HD-VSFG response increases, while the relative contribution of the hydrogen-bonded carbonyls decreases, reflecting cholesterol-induced changes in the membrane H-bond network structure.

We identify two possible explanations for the observed effect. In the first scenario, cholesterol induces conformational changes in PC carbonyl groups, leading to an apparent increase in the population of free carbonyls. However, nuclear magnetic resonance experiments have shown that the structural order parameters of the interfacial regions of the phosphatidylcholine membranes, including the carbonyl region, remain largely unaffected by the presence of cholesterol.^36,58^ Therefore, the structural conformational rearrangements of the hydrogen-bonded and non-hydrogen-bonded carbonyls induced by cholesterol appear unlikely. Alternatively, cholesterol-induced ordering of phospholipid tails results in tighter membrane packing (condensing effect), leading to the expulsion of some water molecules, particularly those located above the headgroup, at the level of the carbonyl groups. This mechanism would indeed increase the relative population of free carbonyls, and we find this explanation more plausible. This effect is less pronounced in the case of DOPC, for which the difference in the hydrogen-bonded and free carbonyl populations in the membrane without cholesterol is smaller than that in the case of DPPC (see Figure 4C and F). This is likely due to the presence of unsaturation in the acyl chains of DOPC, which leads to a higher area per molecule and consequently greater separation between the interfacial moieties of the phospholipids when compared to DPPC (also in the presence of cholesterol), as well as less-effective cholesterol-induced condensing effect.^59,60^ As a result, the ability of cholesterol to effectively perturb the carbonyl region of DOPC is less pronounced compared to DPPC.

It is apparent from Figures 2 and 3C that the band resulting from the sphingomyelin carbonyl stretching coincides to a great extent with the band originating from the cholesterol carbon-to-carbon double-bond stretching. Owing to this overlap, it becomes challenging to trustfully resolve and accurately fit the individual contributions in the mixtures of these two components. Nonetheless, some valuable qualitative observations can still be made.

In the extreme scenario, where cholesterol would not interact with sphingomyelin, one could expect that the Imχ^(2)^ spectra of their mixtures would be a linear combination of their respective vibrational spectra multiplied by their molar fraction in the membrane. We calculated such spectra and determined to what extent they overlap with the experimental spectra. The results of this analysis are shown in Figure S2. In brief, for all three SM/Chol systems, the amplitude of the observed C=O/C=C stretching band is higher than that expected from a weighted sum of Chol and SM. Thus, the results indicate that the HD-VSFG spectra in the mixed SM/Chol systems are certainly not simply additive, indicating the presence of interactions between SM and Chol. The higher peak amplitudes can be either due to an increased membrane density or due to an enhanced orientation of the oscillators (SM C=O and/or Chol C=C).

### Effect of Cholesterol on the Interfacial Water Structure within Model Cell Membranes

**Figure 5:**
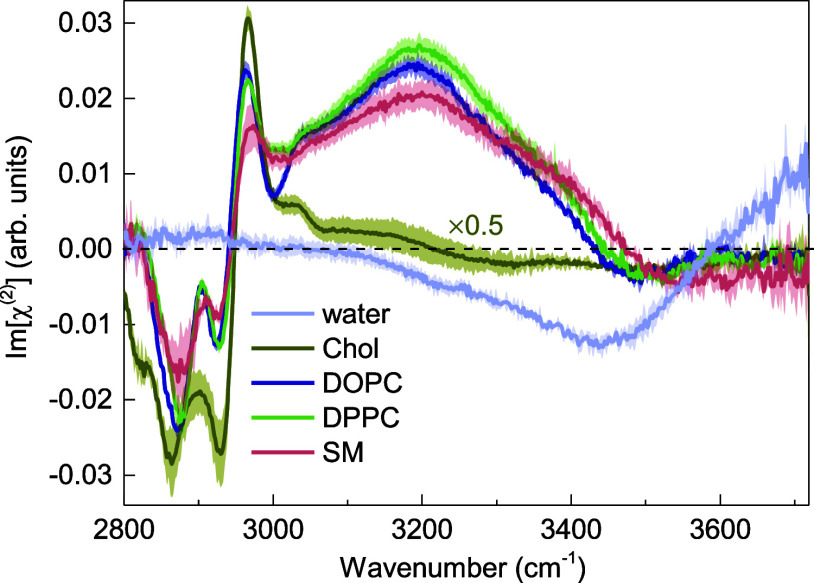
Imχ^(2)^ spectra of neat air–water, DOPC–water, DPPC–water, SM–water, and Chol–water interfaces in the CH and OH stretch vibration region. Curves represent the mean spectra with shaded areas indicating the standard deviation. The spectrum of the Chol monolayer was divided by two for the visualization purposes.

After establishing the impact of cholesterol on the biological water H-bond network from a phospholipid-centric perspective, we proceed to analyze its effects on the interfacial water structure. First, we examine the interfacial water structure within one-component model cell membranes. Figure 5 shows the Imχ^(2)^ spectra of the neat water and one-component phospholipid monolayers on water in the CH and OH stretch vibration region.

The Imχ^(2)^ spectrum of the neat air−water interface (Figure 5, light blue line) is characterized by two bands of opposite signs: a broad negative band with a maximum intensity centered at ca. 3450 cm^−1^ and a narrower positive band at around 3700 cm^−1^. The former is assigned to the OH stretching vibrations of water molecules that donate hydrogen bonds to other water molecules and that have a net orientation with their hydrogen atoms pointing toward the bulk phase (H-down). The higher-frequency band is attributed to the stretching vibrations of the non-hydrogen-bonded (free) OH groups of water molecules, which orient with their hydrogen atoms facing the air (H-up).

For all studied phospholipids (DOPC, DPPC, SM), the Imχ^(2)^ spectrum of the lipid membrane in the CH stretching region (2800−3000 cm^−1^) exhibits characteristic bands arising from the vibrations of methyl groups terminating the hydrophobic chains. The details of the band assignment can be found in the Supporting Information (Note S2).

Phospholipid monolayers clearly rearrange the interfacial water structure. The amplitude of the Imχ^(2)^ signal in the OH stretching region is significantly larger for all three zwitterionic lipids than for the neat water, which is in accordance with earlier studies for zwitterionic lipid monolayers.^15,61^ Regardless of the phospholipid type, the Imχ^(2)^ spectrum shows an intense broad feature with a maximum intensity at around 3200 cm^−1^ with a shoulder band at 3400 cm^−1^. This feature is positive in sign up to approximately 3450 cm^−1^, reflecting H-up oriented hydrogen-bonded water molecules.^62^ In the higher-frequency region, near 3500 cm^−1^, one can observe a relatively weak negative signal that is considered a signature of weakly hydrogen-bonded water molecules buried above the lipid headgroup (H-down oriented).^62−64^ Overall, the Imχ^(2)^ spectrum of SM resembles the spectra of the other two zwitterionic phospholipids. However, two significant differences are observed. First, the doublet feature of the OH stretch band is more pronounced in the case of SM, with the peak at 3400 cm^−1^ being stronger than those in the case of DOPC and DPPC. Second, the negative band near 3500 cm^−1^ extends to the frequency region of the free OH stretch vibration of the water−air interphase. Despite the net zero surface charge of the zwitterionic lipid monolayers, there is a noticeable preferential alignment of interfacial water molecules, reminiscent of a negatively charged interface.^66^ This phenomenon has been hypothesized to be due to the higher charge density and greater reorienting capability of the negatively charged phosphate group compared to the positively charged choline group, favoring an upward orientation of water dipoles.^67^ However, recent HD-VSFG experiments by Dreier et al. on zwitterionic lipids, with reversed positions of phosphate and choline groups, provided evidence that the rationale is different.^15^ This study demonstrated that it is the electric field arising between two oppositely charged groups within the hydrophilic head that governs the net orientation of the interfacial water.^15^ Additionally, Saak et al. have established that the alignment of interfacial water caused by zwitterionic phospholipids is unaffected by ionic screening, thereby evidencing the charge neutrality of such membranes.^68^ Collectively, these findings lead to the conclusion that the probed OH signal in the presently studied model cell membranes originates mainly from water molecules within the primary hydration shell.^68^

**Figure 6:**
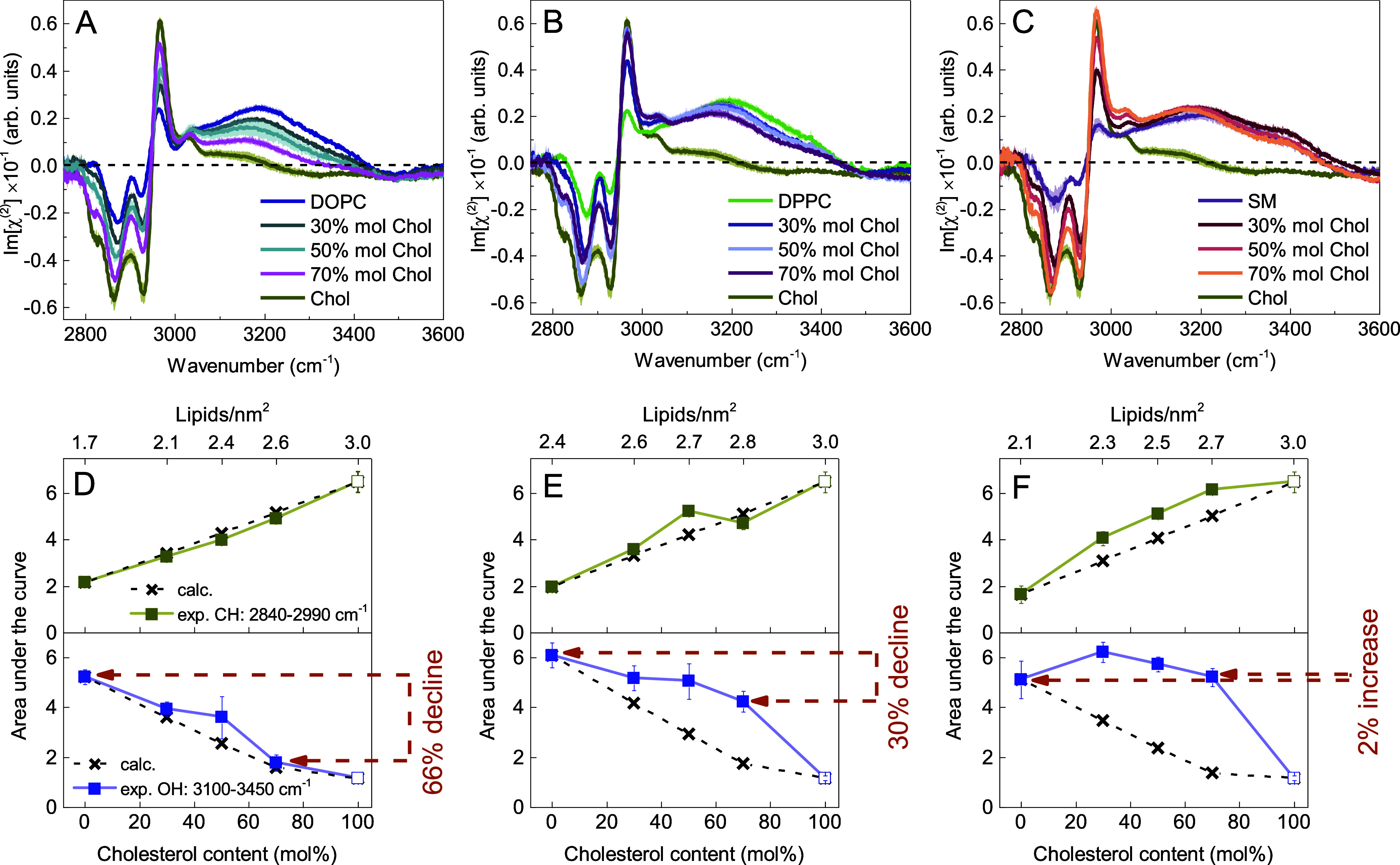
(A–C) Imχ^(2)^ spectra of DOPC, DPPC, and SM monolayers on water, respectively, in the absence and presence of cholesterol at molar fractions of 0.3, 0.5, and 0.7 in the CH and OH stretch vibration regions. The Imχ^(2)^ spectrum of Chol is shown for reference. Curves represent the mean spectra with shaded areas indicating the standard deviation. (D–F) The corresponding area under the curve in two spectral regions (CH and OH stretching) as a function of cholesterol content (bottom *x*-axis) in the monolayers with DOPC, DPPC, and SM as host lipids, respectively. The top *x*-axis indicates lipid densities, calculated using the area per molecule for pure phospholipids and pure cholesterol (at a surface pressure of 40 mN/m), reported in the following references.^45,46,65^ For lipid mixtures, molar fractions were used as weights. The crosses correspond to the spectra calculated as a linear combination of individual components’ spectra, multiplied by their molar fraction in the membrane. The lines connecting the points act as a guide to the eye.

Changes in the Imχ^(2)^ spectra of the lipid−water interfaces in the CH and OH stretch vibration regions resulting from the incorporation of cholesterol into the membranes are depicted in Figure 6. Upon an increase in cholesterol content, the amplitudes of the peaks in the CH stretch vibration region monotonically increase for all three phospholipids. We assign this effect primarily to the following. The Imχ^(2)^ spectrum of a pure cholesterol monolayer exhibits the highest peak amplitudes in the CH stretch vibration region when compared to the phospholipid−water interfaces. This is likely due to the tighter packing (lower area per molecule) and higher number of methyl groups in the cholesterol structure, which increase the effective number of CH oscillators in the probed area. Increasing the molar fraction of cholesterol at the surface thus leads to an increase of the CH signals. The changes of the area under the curve within the 2840−2990 cm^−1^ spectral range for the three phospholipids are shown in the bottom panels of Figure 6.

The response from the nearby water layers is quite different. For DOPC and DPPC host lipids, we observe that the amplitude of the Imχ^(2)^ signal originating from hydrogen-bonded interfacial water molecules in the H-up orientation decreases as the cholesterol content in the monolayer increases. Notably, this change is more pronounced in the case of DOPC, where the area under the curve within the 3100−3450 cm^−1^ spectral range decreases by 66%, compared to 30% in DPPC as the Chol content increases from 0 to 70 mol % (see Figure 6D and E). The decrease of the Imχ^(2)^ response in the OH stretching region with increasing mole fraction of cholesterol, observed for DOPC and DPPC containing monolayers, implies a reduced orientational preference of the membrane-bound water molecules. In SM monolayers, the amplitude of the OH stretch vibration band appears to be virtually insensitive to the cholesterol content (see Figure 6C and F).

The decrease in the interfacial water SFG_ssp_ signal was previously observed in studies involving the zwitterionic deuterated DMPC lipid bilayer upon incorporation of the cholesterol analogue 6-ketocholestanol.^69,70^ The observed effect was attributed solely to membrane dehydration. While it is plausible that the decrease of the Imχ^(2)^ response in the OH stretching region with increasing mole fraction of cholesterol, as observed herein for DOPC and DPPC containing monolayers, may be partially due to membrane dehydration, we consider a reduced orientational preference of the membrane-bound water molecules to be the dominant effect decreasing the water signal. This interpretation is supported by molecular dynamics simulations, which demonstrated a decreased orientational bias of biological water following an increase of the cholesterol content in lipid bilayers composed of PC lipids.^71,72^

To gain further insight into the effect of cholesterol on interfacial water, we compared the experimental responses of mixed phospholipid/Chol membranes with spectra calculated as a superposition of separate contributions based on their molar fraction in the membrane. The corresponding areas under the curve in two spectral regions (CH and OH stretching) are plotted on the bottom panels of Figure 6 (depicted as black crosses). For a direct visual comparison of the experimentally measured Imχ^(2)^ spectrum of the two-component monolayer with the corresponding calculated spectrum, see the Supporting Information. In Figure S3, we provide one exemplary spectral set for each phospholipid.

In the case of DOPC, the measured responses, both from the hydrophobic tails and water, indicate the absence of strong specific interactions between sterol and the unsaturated phospholipid studied (see Figures 6D and S3), consistent with our findings from the carbonyl investigation.

The DPPC/Chol systems show a moderate deviation of the measured response from the hydrophobic tails from the calculated response (13% difference on average). This is likely due to the ordering of phospholipid tails. We also observe that the decrease in the water signal upon adding cholesterol is much less than expected (see Figures 6E and S3). This points at an enhanced orientation of water molecules in the hydration shell of the remaining phospholipid headgroups. We propose that the cholesterol-induced ordering of phospholipid tails entails tighter packing (evidenced also by the cholesterol-induced dehydration of DPPC carbonyl region), resulting in a more vertical and uniform orientation of the lipid terminal methyl groups as well as the headgroups. Collectively, these effects give rise to an increasing signal from oriented water molecules when the Chol concentration increases.

In the case of SM, we observe the highest deviations from the predicted spectra, both in the hydrophobic tails (27% difference on average) and water signals. The amplitude of the OH stretching band does not decline; in fact, it increases slightly (see Figure 6F). It thus appears that in the raft-like SM membrane, the interfacial water remains highly polarized regardless of the cholesterol content. This finding suggests an even more pronounced enhanced packing and a more significant effect of headgroup rearrangement on the orientation of the nearby water molecules, compared to DPPC.

Clearly, our results show that the condensing effect of cholesterol is most pronounced in the case of SM, somewhat weaker in DPPC, and weakest in DOPC systems. In the case of SM, the dilution effect of adding cholesterol appears to be completely absent, which is somewhat unexpected. For glycerophospholipid membranes, the cholesterol hydroxyl group has been recognized to typically reside in the interfacial region of the membrane.^73−75^ For sphingomyelin membranes, the positioning of the sterol OH group might be different. Yasuda et al. demonstrated that the cholesterol-induced lipid tail ordering reaches deeper in the SM membrane when compared to its glycerophospholipid counterpart.^76^ In particular, the rigid fused ring segments of cholesterol were found to be positioned within the central region of the alkyl chains of SM.^76^ The resulting relatively large distance between the hydroxyl group of cholesterol and the amide group of SM would require reorientation of the amide group to enable a direct hydrogen-bonding interaction with the cholesterol hydroxyl group, which was not observed experimentally.^51^ Recent experimental studies further hint in this direction, by showing that neither the conformation of the N-linked long acid chain^51^ nor the orientation of the carbonyl group^77^ of SM is affected by Chol. These findings suggest that a direct interaction between SM’s amide and Chol’s hydroxyl group may not exist. Thus, we conclude that the positioning of the Chol’s hydroxyl group is well above the SM interfacial region (closer to the hydrophobic core). In this connection, it is important to note that sphingomyelin not only possesses hydrogen-bond acceptor groups but also hydrogen-bond donor groups (NH and OH) (see the turquoise arrows in Figure 1). Hence, in an SM membrane, intra- and (direct) intermolecular hydrogen-bonding interactions are present, which distinguish it from glycerophospholipid membranes. Owing to the strong condensing effect of cholesterol, the intermolecular van der Waals contacts between SM molecules are maximized, which may also enhance the intermolecular hydrogen-bond network between SM molecules that pushes cholesterol further toward the hydrophobic region of the membrane.^78^ Thus, in addition to the enhanced packing and headgroup ordering, we attribute the nondecreasing water signal to the strong hydrogen-bond network within the SM, which prevents cholesterol from reaching the interfacial region with its hydrophilic hydroxyl group. Instead, cholesterol is anchored deeper in the membrane interior. As a result, the local preferential orientation of interfacial water molecules is preserved even when the SM membrane contains a large fraction of cholesterol.

## Conclusions

We used heterodyne-detected vibrational sum-frequency generation spectroscopy to study the effect of cholesterol on the structure and hydration of monolayers of 1,2-dipalmitoyl-*sn*-glycero-3-phosphocholine (DPPC), 1,2-dioleoyl-*sn*-glycero-3-phosphocholine (DOPC), and egg sphingomyelin (SM) on water. We probed the response of the carbonyl vibrations of the phospholipids, CH vibrations of the phospholipids and cholesterol, and OH vibrations of water molecules hydrating the lipids. We found that the effect of cholesterol strongly depends on the nature of the host zwitterionic phospholipid monolayers.

**Figure 7:**
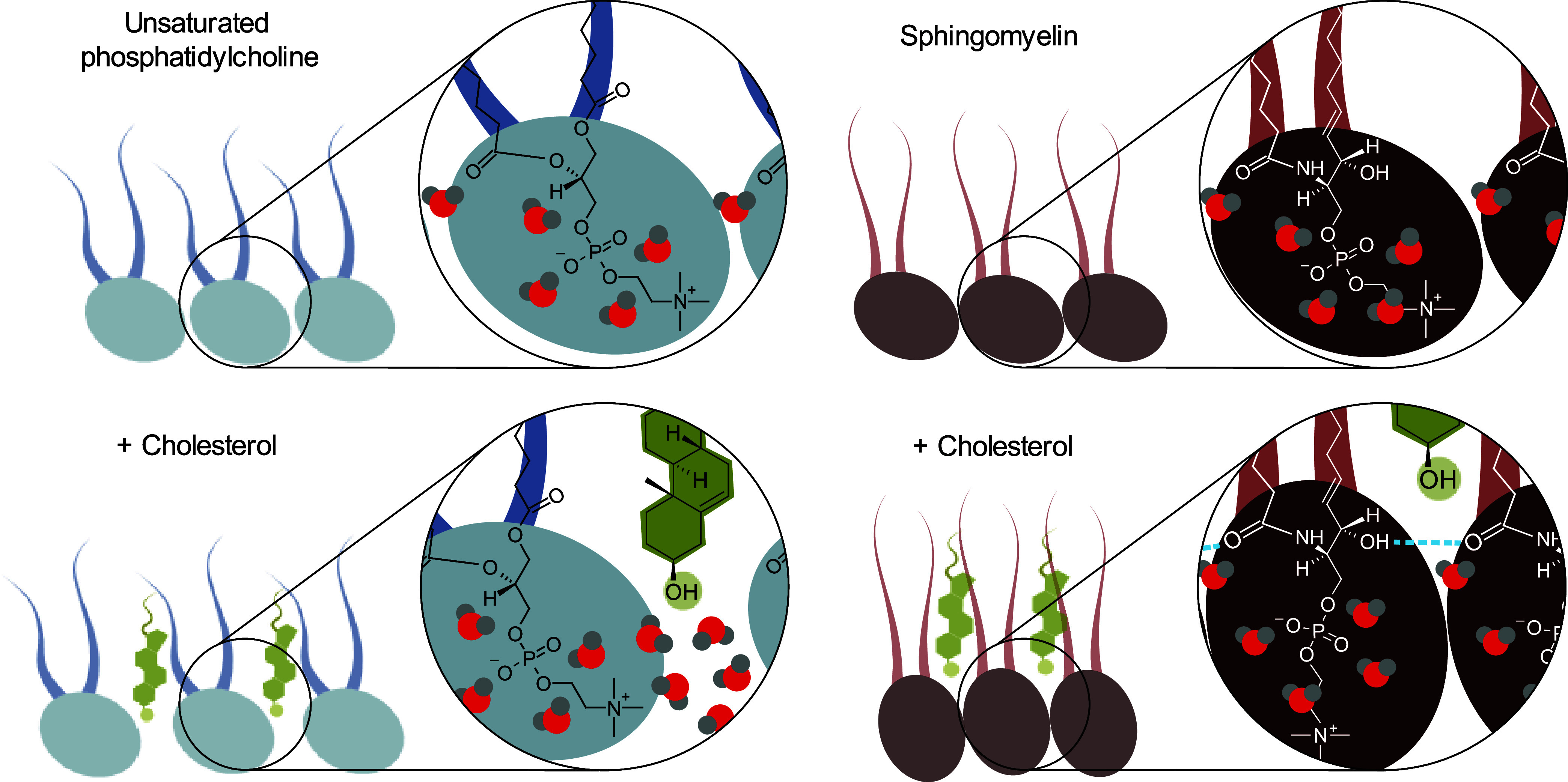
Schematic illustration of the effect of cholesterol on the interlipid interactions and the interfacial water structure. In an unsaturated phosphatidylcholine membrane, cholesterol intercalates into the interfacial region, leading to the protrusion of more bulk-like water molecules between the phosphocholine headgroups. In a sphingomyelin membrane, cholesterol reduces the interlipid distance and facilitates direct intermolecular hydrogen bonding among phospholipids, resulting in the ordering of phosphocholine headgroups and the associated interfacial water molecules.

For the unsaturated phosphatidylcholine DOPC, the responses of the CH and OH vibrations indicate the absence of strong specific interactions between cholesterol and the unsaturated phospholipid tails. Cholesterol intercalates into the membrane, causing increased separation between adjacent phospholipids. Compared to the cholesterol-free membrane, this results in the decline of the population of oriented water molecules associated with the phospholipid headgroups (see the left side of Figure 7).

For the saturated phosphatidylcholine DPPC, we found that the addition of cholesterol leads to a quite strong increase of the CH signal, which indicates an enhanced ordering of phospholipid tails. We also observe a pronounced relative increase of non-hydrogen-bonded carbonyl groups over hydrogen-bonded carbonyl groups, which indicates that the tighter packing is accompanied by expulsion of part of the water molecules, particularly those located above the headgroup, at the level of the carbonyl groups. We also observe that the decrease in the water signal upon adding cholesterol to the layer is much less than expected from the dilution effect. This points at an enhanced orientation of water molecules in the hydration shell of the remaining phospholipid headgroups. We propose that the cholesterol-induced ordering of phospholipid tails entails tighter packing resulting in a more vertical and uniform orientation of the lipid terminal methyl groups as well as the headgroups.

For SM, we observed an even stronger increase in the response of the CH vibration upon adding cholesterol, indicating an even more pronounced enhanced packing and ordering of the hydrophobic tails than those observed for DPPC. We observe that the amplitude of the OH stretching band does not decrease at all upon adding cholesterol despite the dilution effect of adding cholesterol to the layer. It thus appears that the robust intermolecular hydrogen-bond network of SM anchors cholesterol deeper within the nonpolar membrane interior, limiting its ability to disrupt the orientation of interfacial water molecules. Collectively, the two effects act in tandem to preserve the strong orientational bias of interfacial water molecules in raft-like membranes, even at high cholesterol concentrations (see the right side of Figure 7).

Our findings offer the first experimental evidence that cholesterol influences the alignment of biological water, clearly a phenomenon intricately linked to the membrane’s unique composition and the interlipid interactions. The observed strong orientational bias (hence strong membrane dipole potential) in the case of sphingomyelin-rich membranes, even at high cholesterol content conditions, sets sphingomyelin membranes apart from nonraft cell membrane domains and carries potential implications for cellular processes like domain-selective protein binding and membrane fusion events. Membrane dipole potential, originating from the preferential alignment of interfacial water molecules and the anisotropic orientation of lipid dipolar moieties, can reach high values of up to several hundred millivolts,^23^ leading to the formation of a strong, local electric field. It is thus of no surprise that such a dipole potential is very effective in modulating the conformation and function of membrane proteins as well as their distribution and binding affinity.^25,26^ It has been shown that the membrane dipole potential is not homogeneous—on the contrary, it is significantly larger in lipid domains enriched in cholesterol, correlating well with the localization of lipid raft markers within the membrane.^79^ Crucially, the spatial heterogeneity of the membrane dipole potential is interweaved with its temporal variation. Recent studies showed strong dependence of the magnitude of the membrane dipole potential on the stage of the ovary cell (CHO-K1) cycle.^80^ Intriguingly, the measured magnitude of the dipole potential correlated well with the temporal variation of cholesterol in the cell membrane. Our results thus provide a clear molecular-level picture of the origins of composition-specific spatial heterogeneity of the membrane dipole potential, arising from the intricate interactions of cholesterol with membrane lipids and their subsequent effect on the orientational anisotropy of membranes’ hydration layer.

This sheds new light on the role of cholesterol in cell membranes’ biophysical and biochemical activities and, yet again, manifests the structure−function relationship in lipid membranes. We believe these results lay a strong foundation for future research endeavors, both experimental and computational, to unravel the implications of the interplay between cholesterol and the orientation of membrane-associated water molecules in cellular processes.

## Supporting Information Available

Exemplary individual Imχ^(2)^ spectra of the DOPC–water interface at different lipid surface coverages in the OH stretch vibration region; comparison of the averaged experimentally measured Imχ^(2)^ spectra with the calculated ones of SM/Chol monolayers in the C=O stretch vibration region and of DOPC/Chol, DPPC/Chol, and SM/Chol monolayers in the CH and OH stretch vibration regions; description of the origin of the spectral modulation effects; and details of the band assignment in the spectral region 2800−3100 cm^−1^ (PDF)
