# Supplementary material for: Cholesterol Changes Interfacial Water Alignment in Model Cell Membranes
Source: J Am Chem Soc. 2024 Apr 30;146(19):13151–62. doi: 10.1021/jacs.4c00474 (PMC11099968; doi:10.1021/jacs.4c00474)
Supplement: Supplementary file 1 — ja4c00474_si_001.pdf [file ja4c00474_si_001.pdf]

Supporting Information for

Cholesterol Changes Interfacial Water Alignment in Model Cell Membranes

Hanna Orlikowska-Rzeznik,[1*] Jan Versluis,[2] Huib J. Bakker,[2] and Lukasz Piatkowski[1*]

[1]Faculty of Materials Engineering and Technical Physics, Poznan University of Technology, Piotrowo 3, Poznan, Poland
[2]AMOLF, Ultrafast Spectroscopy, Science Park 104, Amsterdam, The Netherlands

Correspondence: hanna.orlikowska@put.poznan.pl (H. Orlikowska-Rzeznik); lukasz.j.piatkowski@put.poznan.pl (L. Piatkowski)

Table of Content:

Supplementary Figures S1–S3

Supplementary Notes S1–S2

Supplementary Figures S1–S3

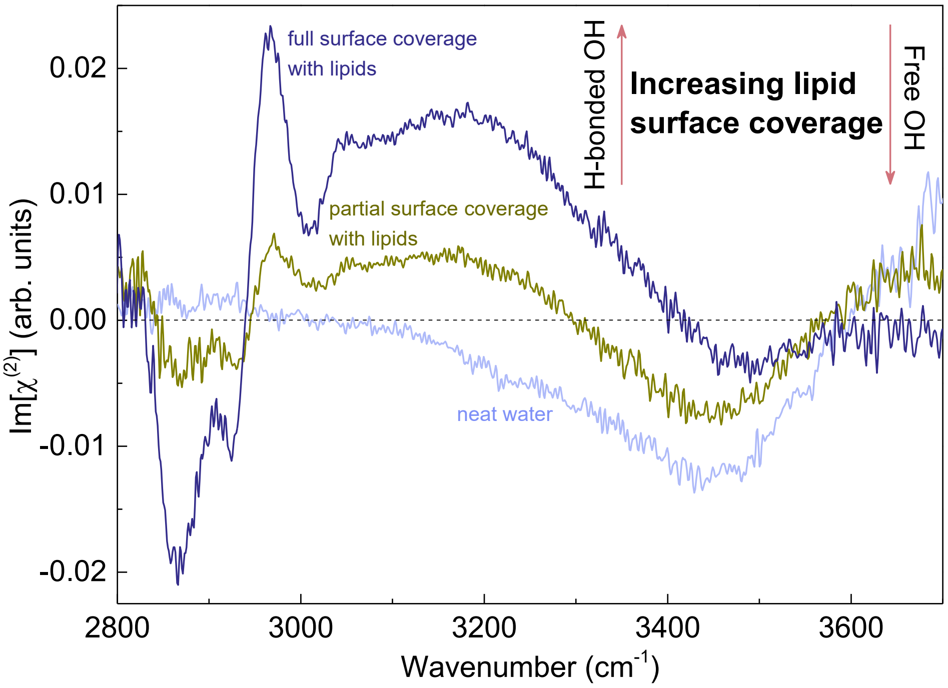

Figure S1. The exemplary Imχ$^{(2)}$ spectra of neat water and the DOPC-water interface at partial and full lipid surface coverage in the CH and OH stretching vibration region. The chosen (complete) lipid surface coverage corresponds to a surface pressure of around 40 mN/m.

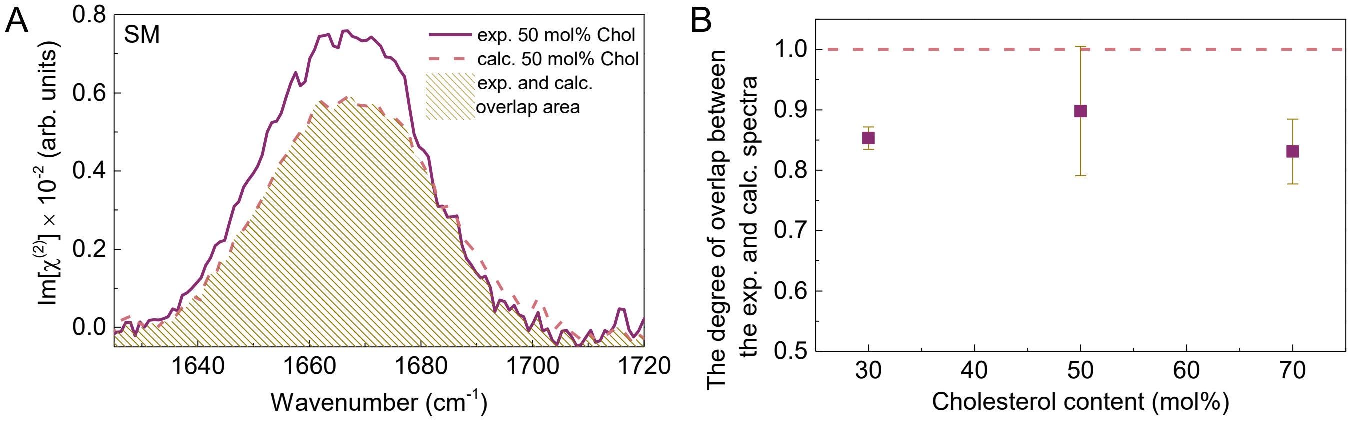

Figure S2. (A) Comparison of averaged experimentally measured Imχ$^{(2)}$ spectrum with the calculated spectrum for a mixed SM/Chol monolayer at cholesterol molar fraction of 0.5 in the carbonyl stretch vibration region. The calculated spectrum results from a linear combination of separate contributions from pure SM and Chol monolayers at ratios equal to molar fractions of lipids, using averaged spectra of the pure lipids. (B) Mean ratio of the overlap area between individual experimental spectra and their corresponding calculated spectra.

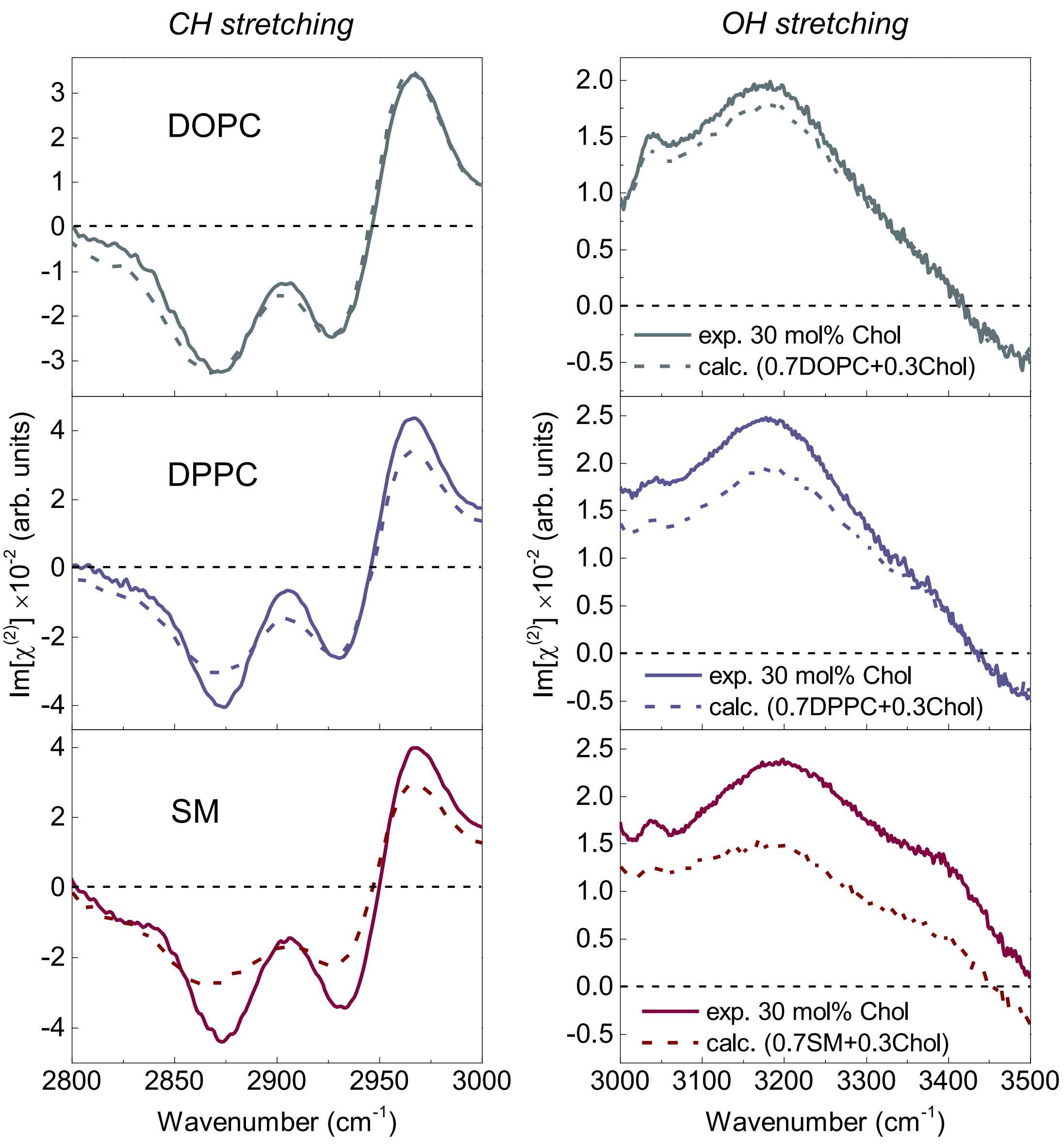

Figure S3. Comparison of the experimentally measured Im$\chi^{(2)}$ spectra of DOPC/Chol, DPPC/Chol, and SM/Chol monolayers at cholesterol molar fraction of 0.3 in the CH (left panels) and OH (right panels) stretch vibration regions with the corresponding calculated spectra as a linear combination of the separate contributions from pure components at their molar fraction. Experimental curves represent the mean spectra. For calculation, the mean spectra of pure DOPC(DPPC) and Chol were used.

Supplementary Notes S1–S2

Note S1

The measured VSFG signal is affected by spectral modulation effects resulting from the etaloning effect in a CCD camera. This effect, caused by light waves passing through the camera and reflecting off the camera rear surface, creates interference patterns that degrade the performance of thinned, back-illuminated CCD cameras. Despite advancements in camera technology aimed at reducing this effect and enhancing data quality, complete elimination remains challenging. The etaloning signal mixes with the heterodyne VSFG signal and cannot be removed by traditional post-processing methods. The correction procedure with the two quartz orientations largely removes the etaloning effect, thereby strongly enhancing the data quality, particularly in the 6 μm spectral region (~1600 cm^{-1}).[1,2]

Note S2

For all studied phospholipids (DOPC, DPPC, SM), the $Im\chi^{(2)}$ spectrum of the lipid membrane in the CH stretching region exhibits the features arising from the vibrations of methyl groups terminating the hydrophobic chains: a negative double-peak feature with maxima at ca. 2870 and 2930 cm^{-1}, assigned to the CH_3 symmetric stretching and the Fermi resonance with its bend overtone, respectively, and a positive band at 2970 cm^{-1}, attributed to the CH_3 asymmetric stretching. The lack of clearly visible signatures from methylene groups (CH_2) is indicative of a well-packed lipid monolayer without isolated gauche defects (~2820 cm^{-1}).[3] The region between 3000 and 3080 cm^{-1} covers the vinyl CH stretching vibration from the double bonds in the acyl chains (present only in the case of DOPC).[4,5] Additionally, it presumably includes the stretching vibrations of the methyl groups on the choline moiety ($N(CH_3)_3$+) weakly interacting with water molecules.[6] In the Chol spectrum, at around 3040 cm^{-1}, a peak can also be distinguished, which most likely originates from the olefinic CH stretching of the double bond structure (C=C-C-H) in the steroid ring structure.[7]

References

(1) Moll, C. J.; Versluis, J.; Bakker, H. J. Direct Evidence for a Surface and Bulk Specific Response in the Sum-Frequency Generation Spectrum of the Water Bend Vibration. *Phys. Rev. Lett.* **2021**, *127* (11), 116001.

(2) Moll, C. J. Bending and Stretching: A Practical Examination of Molecules at Aqueous Interfaces, University of Amsterdam UvA, Amsterdam, 2022.

(3) Backus, E. H. G.; Bonn, D.; Cantin, S.; Roke, S.; Bonn, M. Laser-Heating-Induced Displacement of Surfactants on the Water Surface. *J. Phys. Chem. B* **2012**, *116* (9), 2703–2712.

(4) Liljeblad, J. F. D.; Bulone, V.; Tyrode, E.; Rutland, M. W.; Johnson, C. M. Phospholipid Monolayers Probed by Vibrational Sum Frequency Spectroscopy: Instability of Unsaturated Phospholipids. *Biophys. J.* **2010**, *98* (10), L50–L52.

(5) Maltseva, D.; Gonella, G.; Ruysschaert, J. M.; Bonn, M. Phospholipid Acyl Tail Affects Lipid Headgroup Orientation and Membrane Hydration. *J. Chem. Phys.* **2022**, *156* (23), 234706.

(6) Genova, J.; Petrov, M.; Bivas, I.; Rafailov, P.; Naradikian, H.; Katranchev, B. Fourier-Transform Infrared and Raman Characterization of Bilayer Membranes of the Phospholipid SOPC and Its Mixtures with Cholesterol. *Colloids Surfaces A Physicochem. Eng. Asp.* **2018**, *557*, 85–93.

(7) Kodali, D. R.; Small, D. M.; Powell, J.; Krishnan, K. Infrared Micro-Imaging of Atherosclerotic Arteries. *Appl. Spectrosc.* **1991**, *45* (8), 1310–1317.
